# The prevalence of hepatitis B and C among blood donors at the National Blood Transfusion Center (CNTS) in Burundi

**DOI:** 10.11604/pamj.2018.31.119.14571

**Published:** 2018-10-17

**Authors:** René Kwizera, Amani Moibéni, Farha Shenawy, Mohamed Youssif

**Affiliations:** 1 Laboratory of the National Hospital Clinic Prince Louis Rwagasore, Bujumbura, Burundi; 2 University of Bujumbura Light, Nursing Department Bujumbura, Burundi; 3 University of Burundi, Faculty of Medicine, Bujumbura, Burundi; 4 Mansoura University, Faculty of Pharmacy, Department of Microbiology, Mansoura, Egypt; 5 Mansoura University, Faculty of Pharmacy, Department of Microbiology, Mansoura, Egypt

**Keywords:** Prevalence, HBV, HCV, blood donation

## Abstract

**Introduction:**

in Burundi, blood safety is one of the concerns of the Ministry of Public Health and Fight against AIDS. To this end, all donor blood bags must be screened for hepatitis B and C, that is the reason why the National Blood Transfusion Center (CNTS) has an Enzyme-Linked Immunosorbent Assay (ELISA); better chain to perform these tests properly. This study aimed to highlight the prevalence of hepatitis B and C among blood donors at the National Blood Transfusion Center of Burundi for a period from the January the 1^st^ to the 30^th^ of June 2016.

**Methods:**

this retrospective study was conducted among blood donors (both sexes) aged between 18 years and 50 years. The objective was to collect from the registration registers and the monthly activity reports, the results of the ELISA test obtained after the diagnosis of hepatitis B and C of the blood donors from 1^st^January to 30^th^ June 2016.

**Results:**

in our series of 8,993 samples tested, the prevalence of HBV was 1.04% and HCV was 1.12%. No association VHB- HCV was recorded.

**Conclusion:**

this study showed a low prevalence rate which shows a clear improvement in preventative measures for donor selection and screening tests.

## Introduction

The National Blood Transfusion Center (CNTS in acronym) is a public Institution under the coordination of the Ministry of Public Health and Fight against AIDS. Its headquarters is established in Bujumbura. The Center's overall mission is to organize the Blood Transfusion, establish the quality assurance system to ensure quality control and the safety of the blood products and their derivatives. Hepatitis B is a viral hepatitis due to infection by the hepatitis B virus (HBV) and causing inflammation of the liver. The symptoms of acute disease are essentially inflammation of the liver with or without jaundice and digestive disorders with nausea and vomiting. The chronic carrier has no symptoms but can contaminate his surroundings. In case of chronic active hepatitis, symptoms may be mild fever, fatigue, digestive disorders (nausea and vomiting, abdominal pain), jaundice, dark urine or pale stools. The potential seriousness of hepatitis B consists of the risk of progression to chronic hepatitis B, which can be complicated cirrhosis and a liver cancer, a deadly disease with a very low response rate to the current chemotherapy [1]. Transmission of the virus is through biological fluids and secretions. The main modes of transmission are sexual intercourse, injections among drug users, blood transfusions risk, transmission from mother to child during childbirth and close contact with an infected person. This is a DNA virus responsible for a particular form of viral hepatitis, a disease initially known under the name of serum hepatitis and cause epidemics in parts of the Asia and Africa [2]. Hepatitis B is endemic in China and various other parts of Asia [3]. In countries with low prevalence regions such as the US and Western Europe where less than 2% of the population is chronically infected, drug abuse by injection and unprotected sex are the main transmission routes, although other factors may be important [4]. In moderate prevalence areas, which include Eastern Europe, Russia, and Japan, where 2-7% of the population is chronically infected, the disease is predominantly spread among children. In areas of high prevalence areas like China and South-East Asia, transmission during childbirth is most common, although in other areas of high endemicity such as Africa, Transmission during childhood is an important factor [5]. The prevalence of chronic infection with hepatitis B in areas of high endemicity is at least 8%. Various levels of seroprevalence of HBV are explained by the socio-economic context and vaccination: it allows low prevalences, for example in the Reunion Island (where only 0.7% of the population is affected), so that black Africa or Madagscar often exceeds 15% (e.g. in Madagascar, the prevalence is 16%, due to frequent mother-child transmission and low use of condom, which encourages a frequent sexual transmission). In France, a 2004 survey by the Institute for Public Health showed that 0.65% of adults, or more than 280,000 people are chronic carriers of HBs Ag. Only one in two knew she was HIV positive [6]. Since the rise of vaccination, the prevalence of hepatitis B fell sharply in countries that have implemented universal vaccination strategy [7].

As for hepatitis C, it is an infectious disease transmissible by blood and due to the eponymous virus [8]. It is usually asymptomatic and chronic course. The infection may lead to cirrhosis and liver failure or liver cancer. An estimated 150 to 200 million people worldwide are infected with hepatitis C virus mainly by transfusion that has not been subjected to testing and reuse of needles and unsterile needles [9]. This is a small RNA virus with a linear single-stranded genome of positive polarity. After infection and incubation period of about seven weeks occurs the acute phase of infection [10]. The phase of acute hepatitis C refers to the period from the beginning of the infection to the first six months. This phase is asymptomatic (without symptoms) in 60 to 70% of cases. In a minority of patients, there are non-specific symptoms, such as loss of appetite, tiredness, abdominal pain, flu-like condition, pruritus or jaundice [11]. Transmission of hepatitis C virus (HCV) is parenterally, that is to say, it is transmitted through a route other than that of the gastrointestinal tract. In developed countries, 90% of people infected with chronic infection with hepatitis C were infected by transfusion of blood or blood products not tested or injection drug use or drug inhalation. In developing countries, the primary sources of HCV infection are unsterilized injection equipment and transfusion of blood or blood products tested poorly [11]. Although injection drug use and administration of infected blood or blood products are the most common routes of transmission of HCV infection, any practice, activity or situation that involves blood to blood contact can potentially be a source of HCV infection. The virus can be transmitted sexually but this possibility is rare and generally occurs only when combined with an STD (including HIV), anal sex orduring menstruation, which increases the probability of contact with the blood [11].

In India, in the late 1990s, only 6% of donors were tested for hepatitis C [12]. The transmission of improperly sterilized medical equipment remains a reality in developing countries [13]. In 1999, hepatitis C infected, according to WHO, around 170 million people worldwide [14]. In the United States in the early 1990s, nearly 4 million people were likely affected; 35 000-185 000 new cases occur each year around the US [15]. The co-infection with HIV is common and rates of infection among HIV positive populations are higher. 10 000 to 20 000 deaths per year in the United States are due to hepatitis C; it is expected that the mortality rate increases, as have not been identified all persons who were infected at the time the serological tests for the hepatitis C virus had not yet charged before any transfusion [15]. At the beginning of 2000, a survey showed that prevalence could reach 34% of inmates in California [16]; 82% of subjects who have been diagnosed with hepatitis C have a stay in prison [17], and prison in transmission has been well described [18]. Depending on the region, its prevalence varies from 0.5% to 6% of the population (1% in France, 2% in Japan). In France some 600 000 people have the virus Hepatitis C (1% of the population) over a third are unaware of their condition; between 200 000 and 300 000 people were infected by blood transfusion [19]. There are 5 000 new infections each year, 80% of them being due to drug use [19]. Egypt is the country with the highest prevalence rate of HCV, up to 20% in some areas. One hypothesis is that this high prevalence is linked to a campaign, now discontinued, mass treatment for schistosomiasis, which is endemic in this country [20]. Regardless of the underlying cause of the epidemic, a high rate of HCV transmission continues in Egypt, both of iatrogenic origin and family community transmission [20]. In 2017, according to WHO, there would be 257 million new cases infected with hepatitis B virus (HBV), almost the same number previously estimated (C and B viruses are not related, but both are discrete and persistent for decades and can induce cirrhosis or liver cancer [21]. Together, these viruses are responsible for 96% of deaths from hepatitis and killed 1.34 million people in 2015 alone (about as much as tuberculosis and HIV / AIDS [21].

## Methods

This is a retrospective study which was conducted among blood donors from 18 to 50 years at the National Blood Transfusion Center (CNTS) of Burundi conducted over a period of six months from 1st January to the 30^th^of June 2016. Within the period, the monthly CNTS reports were consulted. All donors were included in this study and no report was excluded. Blood donations were diagnosed routinely for hepatitis B and C, using the ELISA. The latter being an immunoassay, as its primary objective is to demonstrate the presence of antibodies or antigens specific to a disease in a blood sample [22, 23]. The review uses an enzyme called protein that binds to specific component of the disease, and the identification and quantification of this enzyme can be confirmed. For the detection of HBV, direct ELISA technique was used called "sandwich" using the reagent referred MONOLISA HBS Ag PLUS. Monolisa Hbs Ag PLUS is a sandwich-type immunoenzymatic technique in step three using selected monoclonal antibodies for their ability to bind to different subtypes of HBsAg [22, 23]. The solid phase consists of 12 bands of 8 polystyrene wells coated with the first monoclonal antibody. Two other monoclonal antibodies coupled to peroxidase. The test Monolisa HBsAg comprises the following steps: the distribution of the samples and control serum in the wells of the microplate; conjugate distribution using a disposable tip for each sample; Hour incubation; washing and then revelation the enzymatic activity bound to the solid phase by the addition of the substrate; stopping the communication, then reading the optical density at 450/620 nm and interpretation of results [22, 23]. For the detection of HCV, indirect ELISA was used with MONOLISA called ANTI HCV PLUS. Monolisa anti-HCV Plus version 2 based on the use of a solid phase prepared with purified Ag. Three recombinant proteins with E. coli from the selected columns in the nonstructural region (NS3 and NS4) and the structural region of the genome of hepatitis C and a liquid phase (conjugate) comprising anti-human IgG goat antibody purified by affinity chromatography and coupled to peroxidase [23, 24]. The implementation of this technique comprises the following reaction steps: the samples to study and control sera are distributed in the wells. If anti-HCV antibodies are present, they bind to Ag fixed on the solid phase, the anti-human IgG antibody labeled with peroxidase was added after washing. They are fixed in turn to the specific antibody retained on the solid phase, after the removal of unbound enzyme conjugate, the antigen-antibody complex is revealed by addition of substrate, after stopping of the reaction, the reading is done with a spectrophotometer at 450/620 nm. The absorbance measured for the sample to conclude as to the presence or absence of anti-HCV antibodies. The intensity of the color is proportional to the amount of anti-HCV antibody bound to the solid phase [23, 24].

## Results

During the period of 6 months of study, the total number of blood donors was 8993 as indicated in Table 1and Table 2. The gender was not specified in the reports. 94 donors were confirmed positive for VBH as shown by Table 1, a prevalence of 1.04%. Table 2 shows that 101 donors were confirmed positive for HCV and prevalence is at 1.12%. In addition to the positive results that have been achieved dubious results were found 37 cases for HBV (Table 1) and 39 cases for HCV (Table 2). It was noted that in the case of positive and doubtful results, directly the blood bags are destroyed. In total, 271 blood bags were destroyed. In order to respect the spirit of medical ethics, donors checked up positive and doubtful are not abandoned by the center: positive donors are sent a recommendation letter for medical care. And the impaired donors are urged to redo the test after a period of three months and if the result is positive, they are equally given a recommendation letter for medical care. If negative, the donor is assumed not sick and will remain among the center blood donors.

**Table 1:** the prevalence of hepatitis B (the results of the study on the prevalence of hepatitis B, Burundi-CNTS, 2016)

Month	Hepatitis B			
	positive	doubtful	negative	Whole pockets
**January**	23	15	1789	1827
**February**	13	2	1147	1162
**March**	18	3	1240	1261
**April**	20	7	1476	1503
**May**	11	8	1680	1699
**June**	9	2	1530	1541
**Total**	94	37	8862	8993

**Table 2:** the prevalence of hepatitis C (the results of the study on the prevalence of hepatitis C, Burundi-CNTS, 2016)

Month	Hepatitis C			
	positive	doubtful	negative	Whole pockets
**January**	11	10	1806	1827
**February**	23	7	1132	1162
**March**	15	1	1245	1261
**April**	22	10	1471	1503
**May**	13	6	1680	1699
**June**	17	5	1519	1541
**Total**	101	39	8853	8993

## Discussion

The results obtained during this six-month period show that the prevalence of hepatitis B among blood donors in CNTS is 1.04%. Compare this result to the National: In a national survey of the prevalence of hepatitis B in 2002 by CEFORMI CHUK, Burundi, the results were used to deduce the prevalence of hepatitis B. Here are the results obtained: in total, 5569 people were registered, 2660 (47.8%) men and 2909 (52.2%) women. 1051 (18.9%) were in urban areas, 1062 (19.1%) semi-urban area and 3456 (62%) in rural areas. The national prevalence of HBsAg was 4.6% [25]. It was higher in men (5.4%) than women (3.9%) in urban areas, (5.9%) men and (4.3%) women in rural areas [25]. It is among the lowest in sub-Saharan Africa [25]. As for hepatitis C, the results obtained during this six-month study period showed that hepatitis C prevalence among blood donors in CNTS is 1.12%. Compare these results with those obtained in the national same survey, the results were as follows: a total of 5569 people were enrolled, 2660 (47.8%) 2909 men and (52.2%) women. Nationally, the overall prevalence of AcHvC was 8.2% [25]. It was 8.3% of men and 8.1% of women [25]. According strata, the prevalence was 10% in urban areas and 9.1% in the semi-urban and 7.4% in rural [25]. The prevalence of viral markers HBV and HCV at the national level is higher in men than in women [25]. This can be explained by injecting drug which is much more noticeable in men than in women. A prevalence of viral markers HBV and HCV in rural areas is higher, which can be explained by the fact that there is a lack of sufficient information in these areas that the majority of rural residents are illiterate [25].

## Conclusion

The results of our study showed a low prevalence of HBV and HCV at the CNTS-Burundi, reflecting an improvement of preventive measures regarding donor selection and proper use of screening technique that is "ELISA" as a diagnostic technique. The prevalence of viral markers obtained at CNTS during my work are below those of the general population of Burundi reflecting the effectiveness of the selection of blood donors in the center. To keep this rate low, it is necessary for CNTS to continue to make this diagnosis correctly, but also to monitor its donors.

### What is known about this topic

- The use of ELISA in the diagnosis of hepatitis C and B;
- All donor blood packs must be screened for hepatitis B and C.

### What this study adds

- The low prevalence of viral markers HBV and HCV CNTS Burundi;
- The prevalence of viral markers in CNTS is lower than those of the Burundian general population.

## Competing interests

The authors declare no competing interests.
